# Modeling Anthropogenic Pressure with Synthetic Water and Wastewater Media: Growth Response of *Lemna minor* Under a Controlled Chemical Gradient

**DOI:** 10.3390/ma19153178

**Published:** 2026-07-25

**Authors:** Magdalena Nowak, Tomasz Kamizela, Angelika Skorupa, Małgorzata Worwąg

**Affiliations:** Faculty of Infrastructure and Environment, Czestochowa University of Technology, 42-200 Czestochowa, Poland; nowak.magdalena@pcz.pl (M.N.); tomasz.kamizela@pcz.pl (T.K.); angelika.skorupa@pcz.pl (A.S.)

**Keywords:** *L. minor*, anthropogenic pressure, synthetic media, wastewater, river water, caffeine, phytoremediation, nutrient dynamics

## Abstract

Anthropogenic pressure on aquatic environments is currently associated not only with the inflow of nutrients, but also with the presence of municipal micropollutants and complex mixtures of sewage origin. This study aimed to evaluate the growth response of *Lemna minor (L. minor)* and the behavior of the plant–medium system under conditions of a controlled chemical gradient reflecting an increasing degree of anthropogenic transformation of the aquatic environment. The experimental setup included four synthetic media: two variants of river water with varying anthropogenic loads and two variants of synthetic wastewater, one of which was enriched with caffeine as a marker of municipal pressure. The experiment was conducted in two independent 14-day series, analyzing changes in nitrate and orthophosphate concentrations, pH, conductivity, plant morphological condition, and final biomass. The most favorable growth conditions for *L. minor* were found in the river variants, especially in the medium with higher loading, whereas the wastewater variants caused growth inhibition, deterioration in plant condition, and greater instability of physicochemical parameters. In the wastewater media, nitrate concentrations did not show a consistent net decrease, while orthophosphate concentrations remained high. These observations indicate that the measured nutrient forms were not effectively reduced under the tested conditions. However, the processes responsible for their temporal patterns could not be identified from the monitored parameters. The addition of caffeine modified the system’s response, but its effect was not quantitatively consistent across both series. The results confirm the usefulness of synthetic media for modeling anthropogenic pressure gradients.

## 1. Introduction

Anthropogenic pressure is one of the main factors transforming today’s aquatic environment. Surface waters, particularly in urbanized watersheds, are subject to the combined effects of municipal wastewater discharges, nonpoint source runoff, and changes in watershed land use. One of the best-recognized effects of this pressure is eutrophication, defined as the excessive enrichment of water with nitrogen and phosphorus compounds, leading to an increase in primary biomass, algal and cyanobacterial blooms, disturbances in the trophic balance, and deterioration in oxygen conditions [1,2,3]. Although eutrophication remains the primary framework for interpreting anthropogenic changes in water quality, it does not fully capture the complexity of contemporary municipal impacts on aquatic ecosystems [2,4].

In the classical approach, the assessment of anthropogenic pressure on water bodies is based primarily on the analysis of trophic indicators and organic pollution parameters, such as total nitrogen, ammonium nitrogen, nitrates, total phosphorus, orthophosphates, BOD_5_, and COD. These parameters remain fundamental for characterizing biogenic pressure and designing wastewater treatment processes; however, they are insufficient to fully describe contemporary municipal impacts on the aquatic environment. In addition to traditional pollutants, surface waters and municipal wastewater also contain micropollutants, including pharmaceuticals, personal care product ingredients, fragrances, surfactants, artificial sweeteners, and other biologically active compounds [4,5]. Even at trace concentrations, these substances can affect aquatic organisms and alter the ecosystem’s response to concurrent nutrient loading [6,7].

For this reason, modern water quality assessment requires broadening the interpretation beyond classic trophic parameters to include compounds characteristic of municipal pollution. Caffeine, widely recognized as a useful marker of domestic wastewater pollution, is of particular importance here. Its usefulness stems from its widespread consumption, frequent presence in raw and treated wastewater, good analytical detectability, and regular occurrence in receiving waters influenced by sewer systems, stormwater overflows, and domestic discharges [8,9,10]. In environmental studies, caffeine primarily serves as an indicator, as it signals broader municipal pressure associated with the presence of a mixture of other biologically active compounds originating from households and domestic and industrial wastewater [11,12,13]. This perspective is of significant importance in experimental studies involving aquatic organisms. Municipal micropollutants do not necessarily cause acute toxicity alone, but may induce more subtle growth and physiological changes, including inhibition of biomass growth, changes in pigmentation, disturbances in photosynthetic processes, and alterations in organisms’ responses to other components of the medium [6,14,15,16].

In this context, *Lemna minor* (*L. minor*), commonly known as duckweed, is one of the best-studied aquatic macrophytes used both in assessing the toxicity of pollutants and in analyzing the potential for biological water treatment. Its importance stems from its simple morphological structure, rapid growth, vegetative reproduction, minimal space requirements, and high sensitivity to changes in the chemical composition of the aquatic environment [7,17,18]. As an indicator organism, *L. minor* exhibits a rapid and measurable response to environmental stress, including changes in the rate of multiplication, thallus development, content of assimilatory pigments, and final biomass production [6,17,19,20,21]. Duckweed also exhibits documented phytoremediation potential, resulting from its effective uptake of nitrogen and phosphorus compounds, and in some cases also from its role in removing other pollutants, including metals and selected organic compounds [19,20,22,23,24]. The indicator and phytoremediation functions thus complement each other, making *L. minor* a suitable research subject for analyzing media with diverse chemical compositions and varying degrees of anthropogenic pressure [6,7,14].

The importance of *L. minor* is further underscored by its practical application in treatment systems based on floating plants. Duckweed systems have long been considered a solution for removing nutrients from municipal wastewater, aquaculture effluents, agricultural runoff, and other water bodies with elevated nitrogen and phosphorus levels [22,23,25]. Their advantages include low energy consumption, relative operational simplicity, and the ability to combine nutrient removal with biomass production [20,24]. At the same time, the effectiveness of such systems depends heavily on environmental and operational conditions, including the composition of the medium, biomass density, retention time, seasonality, and the nature of the incoming pollutants [22,23,24,25,26,27]. For this reason, controlled laboratory studies remain an essential tool for distinguishing between conditions that promote duckweed growth and factors that induce physiological stress and limit the effectiveness of treatment processes [23,24,25,26].

Synthetic media are particularly useful in this type of analysis; their main advantage is the ability to deliberately control their chemical composition in a repeatable and reproducible manner, while limiting the uncontrolled variability characteristic of environmental matrices [17]. In contrast, real-world water and wastewater are characterized by significant temporal and spatial variability resulting from hydrological conditions, seasonality, ongoing pollutant inflow, and the impact of local sources of anthropogenic pressure. This variability makes it difficult to unambiguously interpret the observed biological responses. Synthetic media, on the other hand, allow for the recreation of precisely defined water quality scenarios, varying in biogenic load, ionic composition, and the presence of selected markers of municipal origin [23,25,28,29,30]. Their usefulness has been confirmed in numerous studies on the growth of *L. minor*, nutrient uptake, and the assessment of phytoremediation potential in controlled model systems. They have been successfully used, among other things, to simulate aquaculture and dairy wastewater, which has enabled a detailed analysis of treatment processes occurring under laboratory conditions [23,26,28,29].

Under real-world conditions, anthropogenic pressure is gradual rather than binary. Water bodies receiving municipal and urban effluents are exposed not to a single factor, but to an increasing complexity of chemical composition [31]. For this reason, the response of aquatic organisms should be analysed not only in relation to a single water quality parameter, but also in relation to a gradient of environmental pressure [7,31,32]. The experimental setup included four synthetic media: two variants of river water with varying anthropogenic loads and two variants of synthetic wastewater, one representing the base wastewater matrix and the other enriched with caffeine as a marker of municipal pressure. Such a setup allows for comparison between river-water variants differing in anthropogenic loading and between wastewater variants with and without caffeine, whilst maintaining comparability between the variants [23,24,28]. However, because no caffeine-only control was included outside the wastewater matrix, the independent effect of caffeine cannot be fully separated from the overall wastewater effect.

This study aimed to assess the growth response of *L. minor* and the behavior of the plant–medium system in synthetic water and wastewater media reflecting an increasing degree of anthropogenic alteration of the aquatic environment. Changes in the concentrations of nitrogen and phosphorus compounds were analysed, and an assessment was made as to whether the presence of caffeine, used as a model marker of municipal pressure, was associated with a modified plant–medium response relative to the corresponding wastewater variant without added caffeine. It was assumed that the response of *L. minor* would depend on the complexity of the medium, and that the introduction of the municipal pressure marker would further alter the course of this response under controlled conditions.

## 2. Materials and Methods

### 2.1. Experimental Set-Up and Incubation Conditions

**Figure 1:**
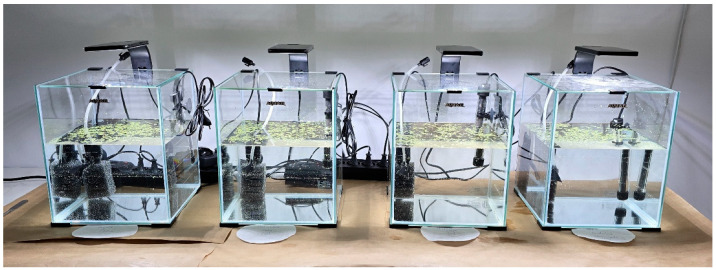
View of the experimental setup with the aquariums used in the experiment.

The experiment was carried out in glass aquariums with fittings and a nominal capacity of 20 L (AQUAEL, Suwałki, Poland) (Figure 1). The working volume in each tank was 14 L. In each aquarium, an internal filter with an aeration function was installed (nominal flow rate 220 L·h^−1^) and operated continuously under identical settings in all variants to ensure comparable mixing and aeration conditions, reduce stagnant zones, and maintain water movement within the plant–medium system. Under such conditions, continuous aeration may also have promoted oxygen-dependent and microbiologically mediated processes, including nitrification, biofilm development, and transformations of nutrient forms; however, these processes were not monitored directly and are therefore interpreted only as potential contributors to the observed changes. The setup was also equipped with an automated LED lighting system controlled by a timer. The light cycle was 8 h·d^−1^, and the medium temperature was maintained within the range of 20–22 °C. The irradiance at the water surface was 23.6–24.8 W/m^2^, which corresponded to approximately 100–120 µmol·m^−2^·s^−1^ PPFD for white LED light. The experiment was conducted in two independent 14-day series performed as separate temporal repeats. Each series constituted a newly established experimental run conducted according to the same predefined experimental protocol, using freshly prepared media and newly introduced plant biomass. These series were treated as independent experimental runs, whereas triplicate measurements at each sampling point represented analytical replicates only.

### 2.2. Preparation of Media and Plant Incubation

Ten grams of fresh *L. minor* biomass was introduced into each aquarium, ensuring a uniform initial biomass across all experimental treatments. The studies were conducted using four different medium variants prepared on the basis of distilled water, which helped to minimize uncontrolled chemical background factors and ensure consistency in composition. Distilled water was used intentionally to create a controlled model system, allowing the composition of each medium to be defined by the added constituents only, rather than by variable background alkalinity, dissolved organic matter, trace components, or microbial load present in natural waters.

The experimental setup included two variants of synthetic river water with varying anthropogenic loads: V1—synthetic river water with a lower load and V2—synthetic river water with a higher load. In addition, two variants of synthetic wastewater were used: V3—synthetic wastewater and V4—synthetic wastewater with the addition of caffeine as a marker of municipal pressure. These media were intended as standardized model wastewater matrices for controlled laboratory comparison and were not designed to reproduce separate real domestic and industrial wastewater streams. The wastewater variants were formulated on the basis of PN-72/C-04550 [33], used here as a technical source for a reproducible model matrix rather than as a regulatory benchmark. Its general composition is comparable to internationally recognized synthetic sewage media described in OECD Test Guideline 303 and the equivalent EU Test Method C.10, although the specific nitrogen and phosphate sources differ [34,35]. The detailed composition and concentrations of the components in the individual variants are presented in Table 1.

### 2.3. Experimental Methodology

During the experiment, the basic physicochemical parameters of the medium were monitored. In situ measurements included temperature, pH, and specific electrical conductivity. In situ measurements were performed using an Elmetron CX-505 multifunction meter (Zabrze, Poland) equipped with a Hydromet ERH-11A pH electrode and a Hydromet CD-201 conductivity probe (Gliwice, Poland)

Turbidity was measured using the nephelometric method with a HACH TL23 laboratory turbidity meter (manufactured in Loveland, CO, USA). At the same time, samples were taken for laboratory analysis of nitrate and orthophosphate concentrations. The analyses were carried out using a spectrophotometric method with a HACH DR 6000 spectrophotometer (manufactured in Düsseldorf, Germany), employing LCK339 cuvette reagent tests for nitrates and LCK348 for orthophosphates, in accordance with the manufacturer’s procedure.

Ammoniacal nitrogen was determined spectrophotometrically using ready-made tests from HACH (method 8155 Powder Pillows—salicylate method). Total nitrogen was determined spectrophotometrically using the Koroleff mineralisation method (peroxodisulphate) and photometric detection with 2,6-dimethylphenol, using HACH cuvette test LCK. Total organic carbon (TOC) was determined in accordance with PN-EN 1484 [36] using a Multi N/C 3100 TOC analyzer from Analytik Jena (manufactured in Jena, Germany), and phosphates by the spectrophotometric method with ascorbic acid in accordance with the methodology described by Hermanowicz et al. (1999) [37].

A key element of the study was the observation of macrophyte morphology, including an assessment of external appearance, the identification of symptoms of chlorosis and necrosis, and an evaluation of the plants’ overall condition depending on the type of medium. Observations were carried out throughout the entire incubation period. Selected observations of macrophyte morphology were carried out using an Olympus BX41 optical microscope (manufactured by Olympus Corporation in Tokyo, Japan).

At the end of each 14-day series, biometric measurements were taken, including the determination of fresh and dry biomass. Wet mass was determined immediately after the end of the experiment, following separation of the biomass from the medium and draining off excess water. The plant material was then dried at 105 °C to constant weight and reweighed to determine the dry weight of the biomass. On this basis, the biomass growth of *L. minor* was assessed according to the type of medium used. The determination of dry weight was carried out in accordance with the procedure described in PN-EN 12880:2004 [38], whilst the selection of wet and dry mass as biometric endpoints was in accordance with the guidelines of OECD 221 [17].

### 2.4. Statistical Analysis

The results of the physicochemical analyses are presented as the mean ± standard deviation from three analytical replicates carried out for each measurement point. To compare the temporal profiles of individual physicochemical parameters between the two independent experimental series, two-way analysis of variance (ANOVA) was performed separately for each medium variant. Experimental series and incubation time were treated as fixed factors, with time considered a categorical variable corresponding to successive measurement points. The series × time interaction was used to assess whether the temporal profiles differed between the two experimental series. Residual normality was assessed using the Shapiro–Wilk test, and homogeneity of variance was assessed using Levene’s test. Statistical analyses were performed using TIBCO Statistica 14.1. Levene’s test did not indicate unequal variances. Non-normal residuals were found for nitrate concentrations in V3 and V4 and for conductivity in V2 and V3. ANOVA was retained because the experimental design was balanced and the homogeneity of variance assumption was met. Results for these four datasets were interpreted with caution. For orthophosphate in V1 and V2, statistical analysis was not performed because concentrations remained below the limit of quantification throughout the experiment. The results of the series × time interaction are presented in the graphs as F and *p* values. A *p* value below 0.05 was considered statistically significant. For all comparisons, the series × time interaction had 5 and 24 degrees of freedom; therefore, only the F and *p* values for this interaction are shown in the graphs.

For each medium variant and each series of the experiment, the net change in the analyzed parameter was also determined as the difference between the final and initial value:

$$\Delta X=X_{14}-X_{1}$$

where ΔX is the net change in the analysed parameter, $X_{14}$ is the value measured on day 14 of incubation, and $X_{1}$ is the value measured on day 1.

The relative change in fresh biomass was calculated in relation to the initial fresh biomass of 10 g:

$$\Delta FM=\frac{FM_{14}-FM_{0}}{FM_{0}}\times 100\%$$

where ΔFM is the relative change in fresh biomass, $FM_{14}$ is the final fresh biomass after 14 days of incubation, and $FM_{0}$ is the initial fresh biomass.

The dry matter share was calculated as:

$$DM_{share}=\frac{DM_{14}}{FM_{14}}\times 100\%$$

where $DM_{share}$ is the proportion of dry matter in the final fresh biomass, $DM_{14}$ is the final dry biomass, and $FM_{14}$ is the final fresh biomass. These indices were used to describe and compare nutrient dynamics, ionic changes, acid–base stability, and biomass response among medium variants.

## 3. Results

The data obtained showed that the response of *L. minor* varied depending on the type of medium used. The most favorable growth conditions were observed in the synthetic river water variants, particularly in V2. In the wastewater variants, reduced biomass growth or biomass loss was accompanied by variable nitrate dynamics, persistently high orthophosphate concentrations, and greater variability in physicochemical parameters. A comparison of the two experimental series showed a consistent overall distinction between the river and wastewater variants. However, the magnitude and temporal course of the responses differed between the series, indicating limited quantitative reproducibility.

**Figure 2:**
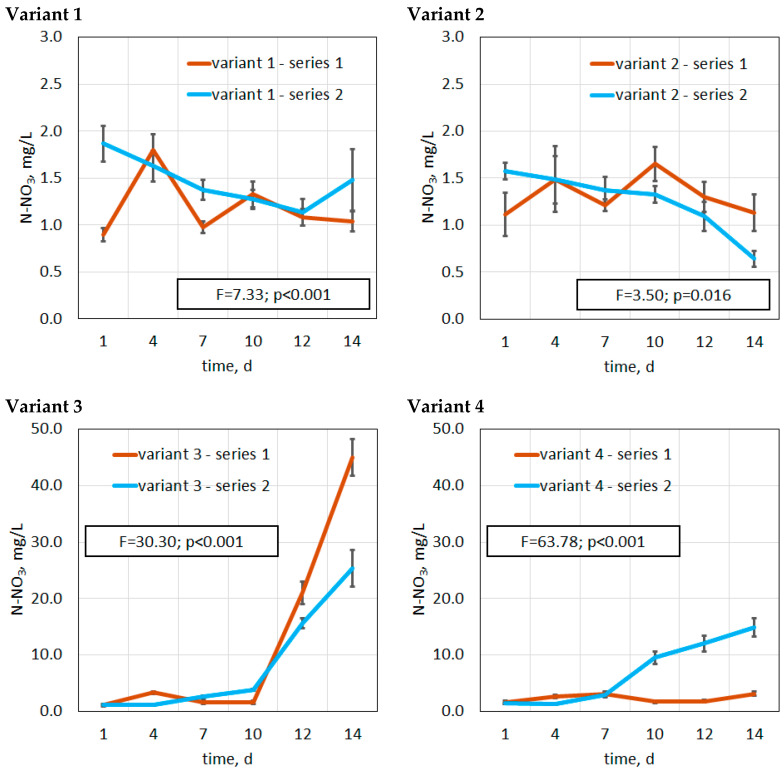
Changes in nitrate concentration over the incubation period in the different medium variants.

Variability in nitrate concentrations was observed, depending on the type of medium used. In the river variants V1 and V2, N-NO_3_ concentrations remained at a relatively low level throughout the incubation period. In Series I, the final N-NO_3_ concentration was 1.0 mg/L in V1 and 1.1 mg/L in V2, whilst in Series II it was 1.7 mg/L and 0.63 mg/L, respectively. A different pattern of changes was observed in the wastewater variants V3 and V4. In variant V3, the N-NO_3_ concentration increased particularly sharply in the final phase of incubation, reaching the highest values of all the systems analysed. In V4, the trend was less clear-cut: in series I, the nitrate concentration remained at a low level, whilst in series II it rose steadily during the second half of the experiment. Thus, no consistent net decrease in nitrate concentration was observed in the wastewater variants. A comparison of the N-NO_3_ concentration curves revealed significant differences between Series 1 and Series 2 in all medium variants. The results of the statistical analysis indicate that, despite the use of the same medium composition and comparable process conditions, the dynamics of changes in nitrate concentration were not fully reproducible between the series. These differences were particularly pronounced in the wastewater variants V3 and V4, which confirms the greater variability in these systems (Figure 2).

**Figure 3:**
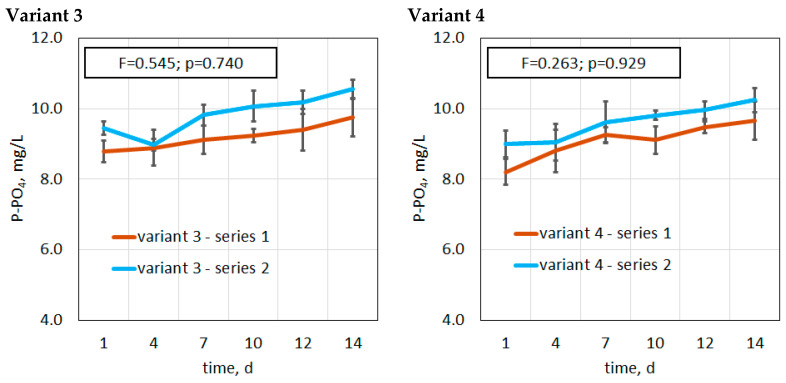
Changes in orthophosphate concentrations during incubation in wastewater variants.

In the river variants V1 and V2, the concentration of orthophosphates remained below the limit of quantification throughout the incubation period. In the wastewater variants V3 and V4, P-PO_4_ concentrations remained high and showed a slight increase during incubation. In Series I, the values increased from 8.78 to 9.76 mg/L in V3 and from 8.82 to 9.66 mg/L in V4. In series II, the corresponding values ranged from 9.45 to 10.55 mg/L and from 8.99 to 10.25 mg/L, respectively. These results do not confirm the effective removal of phosphorus from wastewater media by *L. minor* in the system under investigation. Furthermore, statistical analysis of the curves revealed no significant series × time interaction either in V3: F(5, 24) = 0.545, *p* = 0.740, nor in V4: F(5, 24) = 0.263, *p* = 0.929 (Figure 3). This indicates that the trends in P-PO_4_ concentration were statistically similar between series 1 and series 2.

The specific electrolytic conductivity clearly distinguished between the river and wastewater variants. In the river systems V1 and V2, conductivity values were significantly lower than in the wastewater variants and showed a stable or decreasing trend over the incubation period. In V1, conductivity remained at the lowest level of all variants, whilst in V2 it was higher, which corresponded to the greater mineral load of that medium. In the wastewater variants V3 and V4, conductivity was several times higher than in the river variants. In Series I, it increased from 424.1 to 718.3 µS/cm in V3 and from 413.4 to 807.2 µS/cm in V4. In Series II, the final values were lower than in Series I, but still significantly exceeded the levels observed in synthetic river water. This trend indicates a greater intensity of ionic changes in wastewater media.

**Figure 4:**
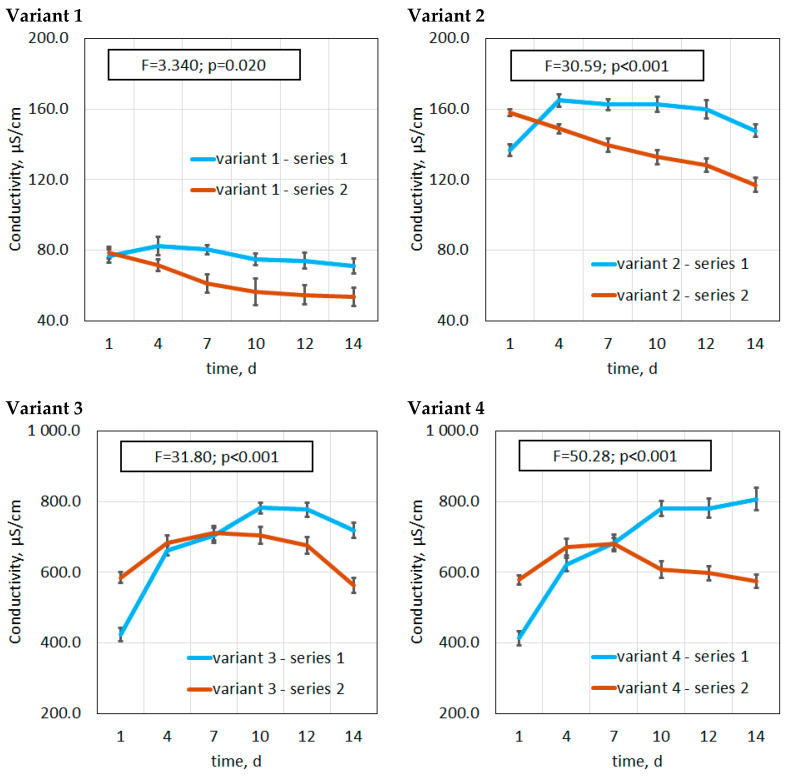
Changes in electrical conductivity during incubation in individual medium variants.

A comparison of the conductivity curves revealed a significant series × time interaction in all medium variants: V1: F(5, 24) = 3.337, *p* = 0.0199; V2: F(5, 24) = 30.587, *p* = 1.15 × 10^−9^; V3: F(5, 24) = 31.797, *p* = 7.74 × 10^−10^; and V4: F(5, 24) = 50.281, *p* = 6.13 × 10^−12^. This indicates that the pattern of conductivity changes between series 1 and series 2 was not fully reproducible. The smallest differences were observed in V1, whilst the most pronounced were in V4, indicating greater process variability in systems with a higher chemical load, particularly in the wastewater variants (Figure 4).

**Figure 5:**
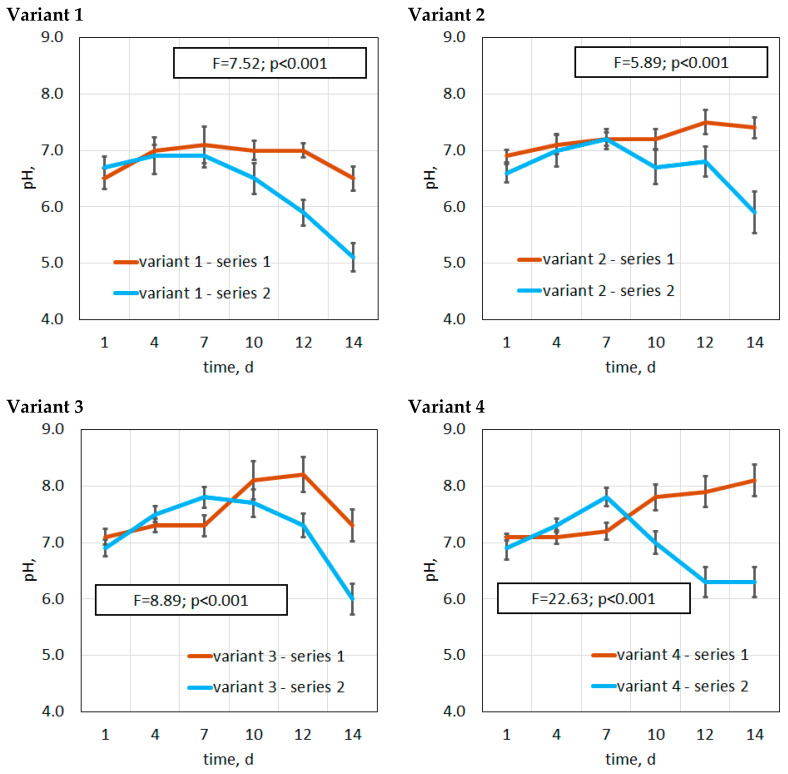
Changes in pH during incubation in individual medium variants. Values in each panel indicate the series × time interaction from two-way ANOVA.

pH values varied over time depending on the medium variant and the experimental series. In the river variants V1 and V2, the pH initially remained close to neutral, whilst in the final phase of series 2, a decrease was observed to approximately 5.1 in V1 and 5.9 in V2, respectively. In the wastewater variants V3 and V4, the pH periodically rose to values of around 8.1–8.2, particularly in series 1, indicating greater variability in acid–base conditions in media with higher organic and mineral loads (Figure 5).

A comparison of the pH curves revealed a significant series × time interaction in all medium variants: V1: F(5, 24) = 7.517, *p* = 0.000228; V2: F(5, 24) = 5.891, *p* = 0.00109; V3: F(5, 24) = 8.888, *p* = 0.0000698; and V4: F(5, 24) = 22.635, *p* = 2.29 × 10^−8^. This indicates that the pH profile was not fully reproducible between series 1 and series 2. The greatest difference in the trend was observed in V4, indicating particularly high variability in acid–base conditions in the effluent variant with added caffeine. The available measurements do not show how much plant activity, organic-matter transformations, or microbial activity contributed to the observed pH changes.

**Figure 6:**
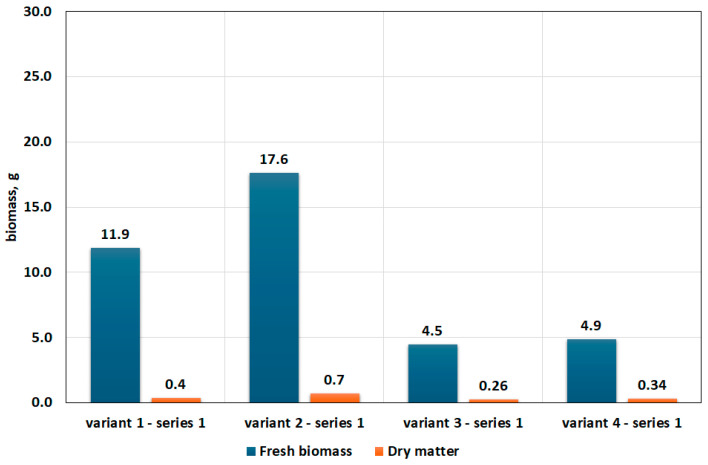
Final fresh and dry biomass of *L. minor* in the tested medium variants in experimental series I.

**Figure 7:**
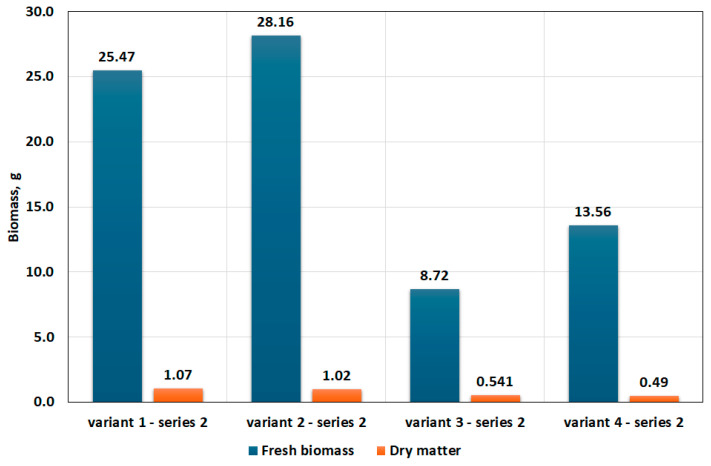
Final fresh and dry biomass of *L. minor* in the tested medium variants in experimental series II.

The final biomass of *L. minor* clearly differed between the river and wastewater variants. In variants V1 and V2, corresponding to synthetic river water, an increase in wet weight relative to the initial value of 10 g was recorded in both series. In series I, the final wet weight was 11.9 g in V1 and 17.6 g in V2 (Figure 6), whilst in series II it reached 25.47 g and 28.16 g, respectively (Figure 7). In both series, variant V2 favored a greater increase in biomass than V1.

In the wastewater variants V3 and V4, the biomass response was markedly weaker and less stable. In Series I, the final wet weight decreased to 4.5 g in V3 and 4.9 g in V4 (Figure 6). In Series II, the final wet weight was 8.72 g in V3, whilst in V4 an increase to 13.56 g was recorded (Figure 7). Despite this improvement, the values obtained in the wastewater variants remained lower than those in the river variants.

Interpretation of the biometric results requires consideration of both wet and dry weight. In Series II, variant V2 achieved the highest wet weight, although its dry weight was slightly lower than in V1. A similar relationship was observed in the wastewater variants: in V4 of Series II, the wet weight was higher than in V3, whilst the dry weight remained lower. This indicates that an increase in wet weight did not always correspond to a proportional increase in dry biomass. Detailed changes in relative biomass and the proportion of dry weight are summarized in Table 2.

A comprehensive analysis of the process parameters confirmed clear differences between the river and wastewater variants (Table 2). In variants V1 and V2, the net changes in nitrate concentrations were negligible or negative, whilst P-PO_4_ concentrations remained below the limit of quantification. This was accompanied by a positive increase in wet weight in all series. In the wastewater variants V3 and V4, however, positive values of ΔN-NO_3_ and ΔP-PO_4_ were observed, along with a weaker and less stable biometric response, particularly in series I. The comparison of the indicators therefore confirms the different behavior of the *L. minor*–medium system in river and effluent media.

**Figure 8:**
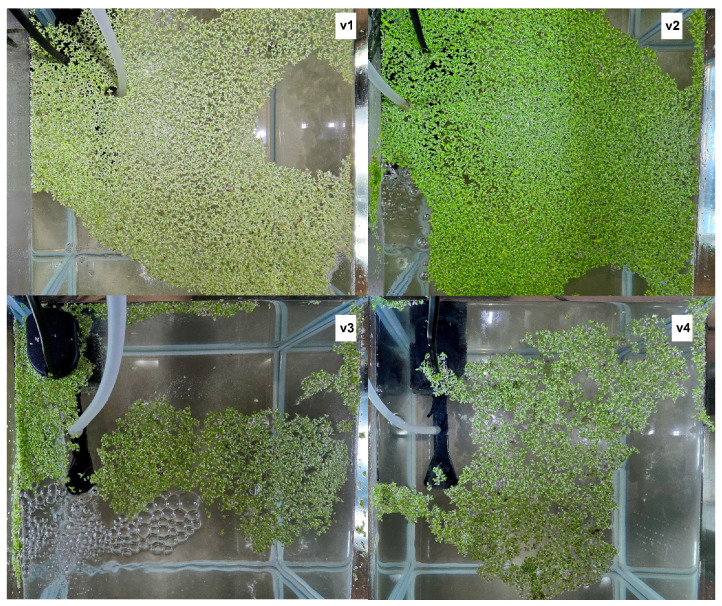
Morphological appearance of *L. minor* in four medium variants on the 14th day of incubation.

In both series of experiments, visual observations were consistent with the physicochemical and biometric results. In the river variants, the plants retained a compact canopy structure, normal green coloration, and uniform coverage of the medium surface, with the most favorable morphological profile observed in variant V2. Variant V1 also favored the maintenance of good condition in *L. minor*, although the degree of surface coverage and biomass density were slightly lower than in V2. A different picture was obtained in the wastewater variants. In V3 and V4, a loosening of the mat structure, poorer distribution of biomass across the surface, and more pronounced signs of deterioration in plant condition were observed (Figure 8).

**Figure 9:**
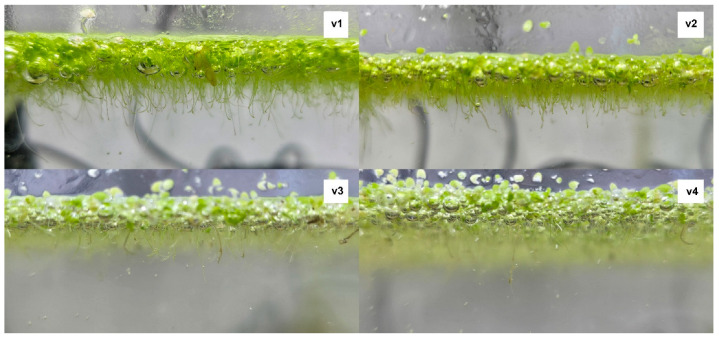
Root system morphology of *L. minor* in the tested medium variants on the 14th day of incubation.

Differences between the variants were also evident within the root system. In the river variants, the roots were longer, more evenly developed, and formed a more organized zone beneath the biomass surface. In the wastewater variants, the root zone was less uniform, and in places shorter roots and a reduction in the compactness of the plant system were observed (Figure 9).

**Figure 10:**
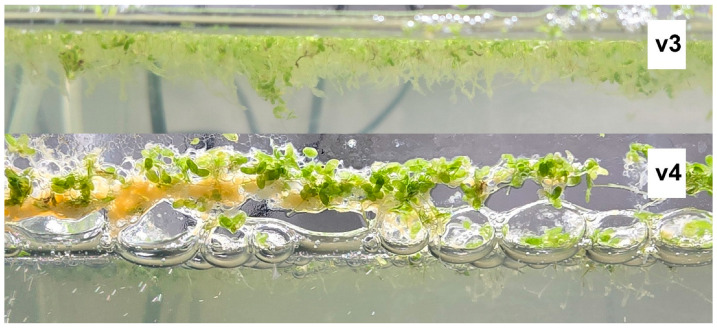
Typical symptoms observed in synthetic wastewater variants include the development of a whitish slime/biofilm in the surface and root zones, as well as a deterioration in the condition of *L. minor.*

In the wastewater variants, a whitish slime was also observed in the surface and root zones, along with signs of stunting and plant weakness. These phenomena occurred alongside cloudiness of the medium, increased conductivity, and persistently high phosphate concentrations. These qualitative observations therefore support the conclusion that the wastewater variants provided less favorable conditions for *L. minor* than the river variants (Figure 10).

**Figure 11:**
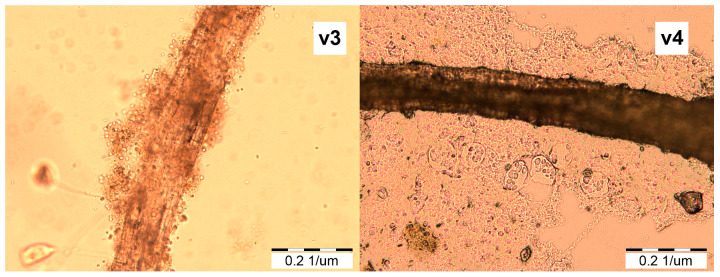
Microscopic image of the white growth observed on the surface of the roots/tissues of *L. minor* in variant 3 and variant 4.

Microscopic examination of the white growth revealed the presence of an irregular layer adhering to the surface of the tissues/roots of *L. minor*, composed of fine particulate and cellular structures, suggesting the development of a biofilm or the accumulation of organic-mineral matter involving microorganisms (Figure 11).

**Figure 12:**
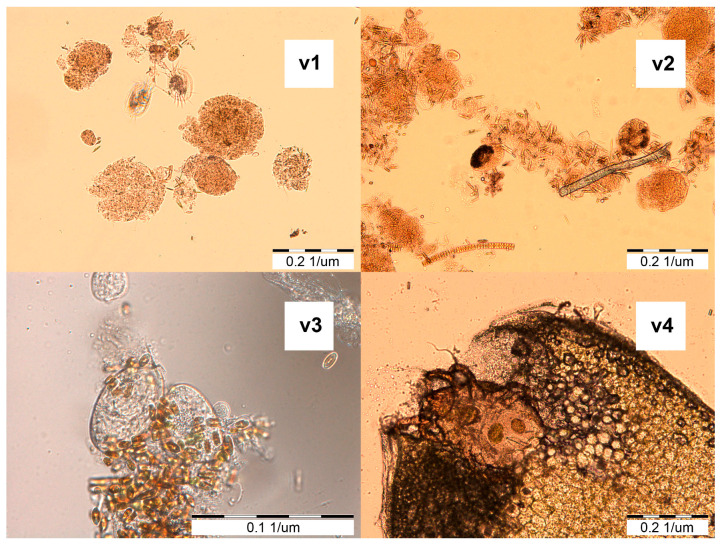
Microscopic image of the bottom sediment in the different medium variants.

Microscopic observations of the bottom sediment revealed the presence of irregular organic-mineral aggregates and associated biological structures (Figure 12). In variants V2–V4, the presence of elongated and elliptical diatom-like structures was particularly pronounced, indicating that the sediment contained not only detrital material but also a significant biological component.

## 4. Discussion

The results obtained indicate that the response of the *L. minor*–medium system was determined by the overall nature of the matrix used, rather than solely by the availability of nitrogen and phosphorus compounds. The key interpretative distinction lay between the synthetic river water variants (V1 and V2) and the synthetic wastewater variants (V3 and V4). The river variants provided conditions favorable for biomass growth and the maintenance of good plant health, whilst the wastewater variants were associated with restricted growth, a deterioration in the condition of *L. minor* and greater physicochemical instability of the medium. This is consistent with reports indicating that the effectiveness of duckweeds depends not only on the presence of nutrients, but also on the organic and ionic load, biomass density and the stability of the entire system [7,24,25].

A key element of the study was the use of the authors’ proprietary synthetic river media V1 and V2. In contrast to the wastewater variants, which were prepared based on the standard concept of synthetic wastewater, the river media were designed to simulate two levels of anthropogenic enrichment of the aquatic environment. This approach should be considered valid, as synthetic media allow for the reduction in the variability typical of environmental samples and the design of controlled exposure scenarios, a method also utilized in other studies involving *L. minor* and model matrices [23,24].

Variant V2 was of particular significance from an interpretative perspective. The higher mineral and biogenic load compared with variant V1 did not inhibit growth; on the contrary, it promoted greater biomass accumulation. This indicates that, within the chemical composition range under consideration, the enrichment of the river environment had a stimulating rather than a stressful effect. This result confirms that *L. minor* can effectively utilize moderately enriched environments, provided that there is no simultaneous overload of the system with organic matter, intensified secondary transformations or destabilization of physicochemical conditions [23,24].

A different response pattern was observed in the wastewater variants. In V3, nitrate concentrations increased, whereas in V4 the nitrate trajectories differed between the two experimental series. Orthophosphate concentrations remained high in both wastewater variants. These observations show that no consistent net decrease in the measured nutrient forms occurred under the tested conditions. The wastewater variants were also characterized by higher conductivity, greater pH variability, and a weaker and less stable biomass response than the river variants. This indicates that nutrient availability alone was not sufficient to ensure favorable conditions for plant growth. Such an interpretation is consistent with previous studies showing that duckweed performance in wastewater systems depends not only on nutrient availability, but also on matrix composition, pollutant load, and overall process conditions [7,25,28]. Several processes may have contributed, including plant uptake, nutrient release, nitrification, organic-matter mineralization, and microbial activity. However, these processes could not be identified because dissolved oxygen, nitrite, time-resolved ammonium nitrogen, and organic-matter indicators were not monitored.

Research [39] has shown that *L. minor* does not grow properly in high-nutrient agricultural effluent under unfavorable chemical conditions of the medium, whilst appropriate pH adjustment can restore its ability to grow and uptake nutrients. This implies that a high concentration of nutrients does not necessarily equate to the medium’s high suitability for phytoremediation. In this context, the results obtained in this study for variants V3 and V4 should be interpreted as an indication that the range of conditions favorable to the growth of *L. minor* has been exceeded.

The river variants were characterized by lower conductivity and less variability in physicochemical parameters, whereas in the wastewater variants, conductivity was many times higher and the pH showed greater fluctuations. Increased turbidity, whitish slime on the roots, and deterioration in plant condition were also observed. These changes could not be linked to a specific biological or chemical process and are treated only as signs of lower stability in the wastewater variants.

Variant V4 deserves a separate comment; it is treated as synthetic wastewater enriched with a municipal pressure marker, rather than as a simple caffeine toxicity test. Caffeine is often used as an indicator of domestic effluent inflow into surface waters [8,40], but in this experiment its effect was not clear-cut. In Series I, variant V4 was associated with an adverse response of the biomass similar to that of V3, whereas in Series II it enabled a partial increase in wet weight. This does not support the interpretation of caffeine as a simple, independent toxic factor with a constant effect. It is more reasonable to treat it as a marker of municipal pressure and a factor modifying the response of the entire sewage matrix, depending on the course of physicochemical and microbiological processes within the system. This is consistent with research on micropollutants, in which the biological effect depends on the concentration of the compound, the duration of exposure, and the co-present components of the medium [6,14,32].

Although the same protocol was used, several responses differed in magnitude and temporal course between the two experimental runs. The source of these differences could not be identified. However, both runs showed the same general distinction between the river and wastewater variants. Individual response values should therefore be interpreted with caution.

Interpretation of nutrient changes was limited by the lack of time-resolved measurements of ammonium nitrogen, nitrite, dissolved oxygen, and organic-matter indicators. Complete nitrogen and phosphorus balances and microbiological analyses were also not available. Therefore, the study describes changes in the measured parameters but does not identify the processes responsible for them.

The results as a whole confirm the usefulness of synthetic media for modelling anthropogenic pressure gradients. In practical terms, the results suggest that *L. minor* may be more useful in systems with moderate loading or in pre-stabilized effluent than in raw matrices characterized by high rates of chemical and microbiological change. This conclusion is consistent with applied studies in which the effectiveness of duckweed depended heavily on the type of waste stream and the operating conditions of the system, rather than solely on the presence of nutrients [25,41]. The synthetic media used, particularly the proprietary river variants V1 and V2, made it possible to capture the transition from growth-promoting conditions to stressful conditions.

## 5. Conclusions

1. The response of the *L. minor*–medium system was clearly dependent on the type of medium used. The most favorable growth conditions were observed in the synthetic river water variants, whilst the wastewater variants limited biomass growth or led to a decline in biomass.
2. The authors’ proprietary synthetic river water media V1 and V2 made it possible to distinguish between lower and higher levels of medium enrichment. Variant V2 favored greater biomass growth than V1, indicating that, within the range studied, the increased mineral and biogenic load did not exceed the threshold inhibiting the growth of *L. minor*.
3. In V3, nitrate concentrations increased, whereas in V4 their temporal pattern differed between the two experimental series. Orthophosphate concentrations remained high in both wastewater variants. Therefore, no consistent decrease in the measured nutrient forms was observed, and the processes responsible could not be identified from the available measurements.
4. The wastewater variants were characterized by greater variability in physicochemical parameters, higher conductivity, greater pH variability, and a deterioration in the morphological condition of the plants. The biomass response was therefore related to the overall state of the plant–medium system, and not solely to the availability of nutrients.
5. The response in V4 differed from that in V3, but the study did not include a caffeine-only medium. Therefore, the observed differences cannot be attributed solely to caffeine or separated from the response of the wastewater matrix.
6. Both experimental runs showed the same general distinction between the river and wastewater variants, but individual responses differed in magnitude and temporal course. These differences should be interpreted with caution.
7. The synthetic media provided a controlled system for comparing the response of *L. minor* along the tested gradient of anthropogenic pressure.
8. Under the tested conditions, *L. minor* performed better in the river-water variants than in the wastewater variants. This suggests that lower matrix loading and greater physicochemical stability may favor plant growth, but this should be verified using real wastewater and pre-stabilized effluents.

## Figures and Tables

**Table 1:** Composition and concentrations of components in the synthetic media used in the individual experimental treatments.

Ingredient	V1 [mg·L^−1^]	V2 [mg·L^−1^]]	V3 [mg·L^−1^]]	V4 [mg·L^−1^]
NaCl (anhyd.)	5.0	15.0	7.0	7.0
CaCl_2_·2H_2_O	7.9	10.5	4.7	4.7
MgSO_4_·7H_2_O	7.3	12.1	2.0	2.0
NaHCO_3_ (anhyd.)	4.0	5.0	–	–
Na_2_SO_4_ (anhyd.)	1.0	2.0	–	–
KNO_3_ (anhyd.)	1.0	2.5	–	–
NH_4_Cl (anhyd.)	–	1.25	20.0	20.0
KH_2_PO_4_ (anhyd.)	0.5	1.0	20.0	20.0
Na_2_SiO_3_·5H_2_O	1.2	1.2	–	–
Peptone (AR)	2.0	8.0	–	–
Humic + fulvic acids (1:1)	2.0	5.0	–	–
FeCl_3_·6H_2_O	0.27	0.27	–	–
MnSO_4_·H_2_O	0.09	0.09	–	–
ZnSO_4_·7H_2_O	0.14	0.14	–	–
Nutrient broth	–	–	105.0	105.0
Peptone (Casein)	–	–	156.0	156.0
K_2_HPO_4_ (anhyd.)	–	–	50.0	50.0
Caffeine	–	–	–	1.0
Ammonium nitrogen, N-NH_4_	LOQ	0.33 ± 0.02	5.24 ± 0.23	5.24 ± 0.22
Total nitrogen, TN	0.48 ± 0.024	1.97 ± 0.09	39.14 ± 1.96	39.43 ± 1.98
Total phosphorus, TP	0.11 ± 0.006	0.23 ± 0.01	13.44 ± 0.67	13.44 ± 0.65
TOC	2.00 ± 0.09	6.50 ± 0.33	115.80 ± 4.8	116.29 ± 5.2

**Table 2:** Process indicators for the *L. minor*–medium system.

Series	Variants	ΔN-NO_3_ [mg/L]	ΔP-PO_4_ [mg/L]	Δconductivity [µS/cm]	ΔpH	Fresh Biomass Change [%]	Dry Matter Share [%]
I	V1	+0.14	<LOQ	−5.50	0.00	+19.0	3.36
	V2	+0.02	<LOQ	+11.13	+0.50	+76.0	3.98
	V3	+43.90	+0.97	+294.20	+0.20	−55.0	5.78
	V4	+1.56	+1.46	+393.80	+1.00	−51.0	6.94
II	V1	−0.39	<LOQ	−25.27	−1.60	+154.7	4.20
	V2	−0.93	<LOQ	−41.03	−0.70	+181.6	3.64
	V3	+24.24	+1.10	−22.50	−0.90	−12.8	6.21
	V4	+13.47	+1.26	−4.00	−0.60	+35.6	3.61

## Data Availability

The original contributions presented in this study are included in the article. Further inquiries can be directed to the corresponding author.

## Abbreviations

The following abbreviations are used in this manuscript:

AR	Analytical Reagent Grade
BOD	Biochemical Oxygen Demand
COD	Chemical Oxygen Demand
FA	Fulvic Acid
HA	Humic Acids
LOQ	Limit of Quantification
TN	Total Nitrogen
TP	Total Phosphorus
TOC	Total Organic Carbon
V1	Synthetic river water with a lower load
V2	Synthetic river water with a higher load
V3	Synthetic wastewater
V4	Synthetic wastewater with added caffeine as an indicator of municipal pressure
